# Urban Housing Preferences and Public Acceptance of Modular Housing Across Different City Typologies in Indonesia

**DOI:** 10.12688/f1000research.185553.2

**Published:** 2026-11-03

**Authors:** Alfandi Rizki Pratama R, Ludfi Djakar, Kartika Puspa Negara

**Affiliations:** 1 Brawijaya University Department of Civil Engineering, Malang, East Java, Indonesia

**Keywords:** modular housing, housing acceptance, relative weight analysis, confirmatory factor analysis, urban typology, Indonesia

## Abstract

**Background:**

Modular housing has the potential to be a strategic solution to the housing affordability crisis in Indonesia’s urban areas. However, the extent of public acceptance of this approach is not yet well understood, particularly across diverse urban typologies.

**Methods:**

This study identifies and compares the factors influencing the acceptance of modular housing in four cities with different urban typologies Jakarta, Surabaya, Sidoarjo, and Malang, through a structured survey of 300 respondents selected using a combination of purposive and quota sampling. The research instrument was validated through Confirmatory Factor Analysis (CFA), yielding AVE and CR values above the required thresholds, with acceptable model fit across all cities. Relative Weight Analysis (RWA) was applied to identify the relative contributions of seven predictors to the acceptance of modular housing.

**Results:**

The results show that Construction & Housing Quality is the most consistent and dominant predictor of acceptance, ranking first in three of the four cities (Jakarta, Surabaya, and Malang), while Sidoarjo was the sole exception, with Financial Considerations ranking first. Other preference hierarchies varied systematically according to urban typology: Location & Accessibility was the strongest secondary factor in Jakarta; Ownership & Flexibility stood out in Sidoarjo; Construction & Housing Quality was most concentrated in Malang; and Surabaya exhibited a holistic and relatively balanced distribution. Developer Credibility consistently ranked as the factor with the lowest weight in nearly all cities.

**Conclusion:**

Although the acceptance of modular housing is influenced by a number of similar preference factors, the priority of these factors differs according to urban typology. These findings introduce the concept of typology preference alignment as a new analytical framework and have practical implications for developers and policymakers in the development of the modular housing sector in Indonesia.

## Introduction

The ever-increasing urban population growth has become one of the main challenges in providing adequate housing in various countries, including Indonesia. Rapid urbanization is driving up housing demand, while land availability in urban areas is increasingly limited and construction costs continue to rise. These conditions call for a more efficient, faster, and sustainable approach to housing development. In recent years, modular housing has emerged as a promising alternative because it can accelerate construction, improve resource efficiency, and support more sustainable development ( Parisi & Donyavi, 2024; Saad et al., 2025; Kamali et al., 2025).

Modular housing is a construction system based on off-site construction, in which building components are manufactured in a factory before being assembled at the project site. Various studies indicate that this approach can reduce construction time, improve building quality through a more controlled production process, minimize material waste, and lower environmental impact compared to conventional construction methods ( Kamali & Hewage, 2016; Ali et al., 2023; Zohourian et al., 2025). Nevertheless, the success of modular housing implementation is determined not only by technical advantages but also by the level of acceptance among the public as end-users. Perceptions regarding building quality, safety, design flexibility, aesthetics, and the image of modular homes have been shown to influence the public’s willingness to adopt this concept ( Steinhardt & Manley, 2016; Lee et al., 2019; Kim et al., 2023; Brickell et al., 2023).

Acceptance of modular housing is closely linked to the public’s preferences in choosing a home. Housing preferences reflect an individual’s evaluation of housing attributes, such as location, price, accessibility, building quality, environmental quality, safety, and supporting facilities. Various studies indicate that these preferences are influenced by demographic characteristics, socioeconomic conditions, and the context of urban development ( Takabatake & Hasegawa, 2022; Rajabi et al., 2024). In Indonesia, public preferences for housing are also influenced by household characteristics, regional development levels, and socioeconomic conditions that vary across regions ( Yanti et al., 2020; Adianto et al., 2023; Analisa & Okada, 2023). Therefore, the level of acceptance of modular housing is likely to vary according to the characteristics of the city where people live.

Although research on modular housing has grown rapidly over the past decade, most studies still focus on technical aspects, such as construction efficiency, sustainability, productivity, manufacturing processes, and building performance throughout its life cycle ( Kamali & Hewage, 2016; Ali et al., 2023; Saad et al., 2025; Zohourian et al., 2025). In contrast, research linking urban housing preferences to public acceptance of modular housing remains relatively limited, particularly studies that account for differences in characteristics across urban regions. In fact, variations in a city’s physical, social, and economic conditions have the potential to shape different housing preferences, meaning that the level of acceptance of modular housing cannot be assumed to be uniform.

This gap becomes increasingly significant when examined in the context of Indonesia as a developing country facing high rates of urbanization, a large housing backlog, and regional disparities in housing supply. Unlike developed countries, which already have relatively well-established construction industrialization systems and housing markets, Indonesia still faces constraints such as limited urban land, rising land prices, varying levels of purchasing power among the population, and regional disparities in infrastructure quality. These conditions make Indonesia a relevant context for evaluating how the public perceives modular housing as a more efficient and sustainable alternative for housing provision.

Furthermore, Indonesia has a highly diverse range of city types, ranging from metropolitan areas, large cities, and medium-sized cities to educational hubs, each with distinct characteristics in terms of population density, economic activity, land prices, infrastructure provision, and housing market dynamics. These differences in characteristics are expected to influence the public’s priorities when evaluating housing attributes and, in turn, shape the level of acceptance of modular housing. However, research in Indonesia generally still addresses housing preferences and modular construction separately, while studies that comparatively analyze the relationship between urban housing preferences and public acceptance of modular housing across various urban typologies remain very limited ( Maryati et al., 2021; Carissa et al., 2025; Yanti et al., 2020; Adianto et al., 2023; Analisa & Okada, 2023). This gap is important to address because effective modular housing development strategies require an understanding of differences in public preferences across each urban typology.

Given this gap, this study aims to analyze the relationship between urban housing preferences and public acceptance of modular housing across various urban typologies in Indonesia. This study identifies the housing attributes that influence public acceptance of modular housing and compares the extent of these attributes’ influence across cities with different urban characteristics. With Indonesia serving as a representative of a developing country with diverse urban typologies, this study is expected to contribute to the development of the literature on the acceptance of modular housing, while also providing empirical evidence that can serve as a basis for governments, urban planners, and developers in formulating housing provision strategies that are more contextual, adaptive, and sustainable.

## Literature review

### Modular housing

Modular housing is an off-site construction approach that involves manufacturing building components in the form of modules in a factory setting before they are transported and assembled at the project site. Unlike conventional construction methods, where most of the process takes place on-site, modular housing shifts production activities to more controlled manufacturing facilities, thereby enabling improvements in efficiency, productivity, and construction quality ( Kamali & Hewage, 2016). Each module is designed as a standalone unit or can be combined with other modules to form larger and more complex buildings according to user needs ( Lawson et al., 2014). These characteristics make modular housing one of the most widely used innovations in modern housing development.

Various previous studies have shown that modular housing offers several advantages over conventional construction methods. Production carried out in a factory environment allows for better quality control, such as reducing material waste, improving workplace safety, and minimizing dependence on weather conditions during the construction process ( Ali et al., 2023; Kamali et al., 2025). Furthermore, the modular approach has proven capable of significantly accelerating project completion. Zohourian et al. (2025) reported that the implementation of volumetric modular construction can reduce construction time by up to approximately 50% compared to conventional methods. These advantages make modular housing increasingly relevant for meeting housing supply needs in urban areas experiencing rapid population growth.

In addition to offering benefits in terms of efficiency and productivity, modular housing is also viewed as an approach that supports sustainable development. Standardized production enables more efficient use of materials, a reduction in construction waste, and better control of energy consumption compared to conventional construction ( Parisi & Donyavi, 2024; Saad et al., 2025). Nevertheless, the success of implementing modular housing is not determined solely by its technical advantages. As a housing product, public acceptance of this concept is also a critical factor in determining its successful adoption. Therefore, in addition to understanding the characteristics of modular housing, it is important to examine how the public perceives and accepts this housing concept.

### Modular housing acceptance

Modular housing acceptance refers to the extent to which the public is willing to accept, use, or choose modular homes as a housing alternative compared to homes built using conventional construction methods. In the context of housing innovation, public acceptance is a critical factor because the success of a technology is determined not only by its technical advantages but also by users’ perceptions and beliefs regarding the benefits it offers. Various studies indicate that the public tends to accept modular housing when they understand the benefits it provides, such as faster construction times, cost efficiency, better construction quality, and higher potential for environmental sustainability ( Steinhardt & Manley, 2016; Lee et al., 2019).

Nevertheless, the level of acceptance of modular housing still varies significantly across countries and social groups. Research in South Korea indicates that the construction of modular homes for public rental housing has received a positive response because it accelerates housing provision and improves construction cost efficiency ( Lee et al., 2019). Another study in Hong Kong found that design flexibility is one of the key factors driving public acceptance, as it allows residents to customize the layout and characteristics of their homes to suit their needs ( Pan et al., 2023). These findings suggest that the public considers not only economic aspects but also how well a home aligns with their needs and lifestyle.

On the other hand, various studies also indicate that negative perceptions regarding quality, durability, aesthetics, and comfort remain major barriers to the adoption of modular housing. In Australia, for example, some members of the public still associate modular homes with temporary structures or simple buildings of lower quality compared to conventional homes ( Brickell et al., 2023). Similar conditions are also found in several developing countries, including Indonesia, where public understanding of modular construction technology remains relatively limited ( Carissa et al., 2025). Therefore, acceptance of modular housing is influenced not only by the characteristics of the technology itself but also by how people evaluate the housing attributes they consider important when choosing a home.

### Urban housing preferences

Urban housing preferences refer to the preferences of individuals or households regarding various attributes considered when selecting housing in urban areas. These preferences reflect the process of evaluating housing characteristics deemed capable of meeting the functional, economic, and social needs of residents. In the housing literature, attributes that are often considered include location, price, accessibility, building quality, environmental conditions, supporting facilities, home size, and spatial flexibility ( Takabatake & Hasegawa, 2022; Rajabi et al., 2024). Differences in priorities regarding these attributes lead to variations in people’s decisions when choosing a type of housing.

Various studies indicate that housing preferences are influenced by socioeconomic characteristics and the environmental conditions of the living area. Households with different income levels tend to have varying priorities regarding price, building quality, and available amenities. Additionally, urban characteristics such as population density, transportation access, land prices, and infrastructure availability also influence how people evaluate a housing unit ( Takabatake & Hasegawa, 2022). Thus, housing preferences cannot be viewed as a universal factor because they are influenced by different regional contexts and societal conditions.

In the Indonesian context, several studies indicate that location, accessibility, environmental quality, housing prices, and supporting facilities are factors that are often the primary considerations in housing selection ( Yanti et al., 2020; Adianto et al., 2023; Analisa & Okada, 2023). However, the level of importance of each attribute may vary across cities depending on the characteristics of their regional development. Therefore, understanding housing preferences is crucial for explaining why communities exhibit varying levels of acceptance toward the concept of modular housing, particularly in cities with diverse urban characteristics.

### City typologies and housing preferences

Urban typology describes the classification of urban areas based on specific characteristics, such as city size, population density, level of economic development, availability of infrastructure, and the area’s function within the urban system. These differing characteristics influence patterns of community activity, spatial needs, and housing market dynamics. In the context of urban planning, understanding urban typology is crucial because each type of city faces distinct development challenges and needs, including the provision of adequate and affordable housing.

Various studies indicate that people’s housing preferences are influenced by the urban context in which they live. In metropolitan cities with high population density and expensive land prices, residents tend to place greater emphasis on accessibility, spatial efficiency, and affordability. Conversely, in relatively smaller cities, factors such as environmental quality, home size, and neighborhood comfort often take precedence ( Rajabi et al., 2024; Takabatake & Hasegawa, 2022). These differences indicate that urban characteristics play a role in shaping residents’ preferences regarding housing attributes.

Indonesia is a country with a highly diverse range of urban typologies, ranging from metropolitan cities like Jakarta and major cities like Surabaya to satellite cities and developing medium-sized cities, each with distinct economic and social characteristics. This variation has the potential to produce different patterns of housing preferences, meaning that the level of acceptance of modular housing may also vary from city to city. Therefore, an analysis based on urban typology is necessary to understand whether the housing attributes that influence the acceptance of modular housing are consistent or differ across different urban contexts.

### Theoretical framework

This study is based on the assumption that public acceptance of modular housing is the result of individuals’ evaluations of various housing attributes considered important in the housing decision-making process. In the context of choosing a place to live, individuals tend to evaluate housing options based on a set of attributes that include location and accessibility, financial considerations, construction and building quality, environmental quality, facilities and spatial layout, developer credibility, as well as flexibility in ownership and building development. This attribute-based evaluation approach is rooted in consumer preference theory, which states that the higher the alignment between the offered attributes and the needs and expectations of prospective residents, the greater the tendency to accept a particular housing option ( Yanti et al., 2020; Adianto et al., 2023). In the specific context of modular housing, this attribute evaluation is influenced by the public’s relatively limited familiarity with the concept of modular construction, where perceptions of quality and flexibility serve as critical differentiating factors compared to conventional housing ( Oluwunmi & Coker, 2024; Phillips et al., 2016).

This study identifies seven housing attribute constructs as the primary determinants influencing modular housing acceptance: Location & Accessibility, Financial Considerations, Construction & Housing Quality, Environmental Quality, Residential Facilities & Space, Developer Credibility, and Ownership & Flexibility. Acceptance of modular housing is further measured through three indicators representing the dimensions of adoption intent and preference: preference for modular homeownership, intention to purchase at an affordable price, and intention to apply for a mortgage to acquire modular housing. Collectively, these three indicators reflect behavioral intentions that progress from cognitive preferences toward financial commitment, consistent with the hierarchy of innovation adoption outlined in the literature on housing acceptance ( Yaacob et al., 2025; Akbar et al., 2023). Thus, modular housing acceptance in this study is viewed as a representation of the public’s intention to accept, choose, and adopt the concept of modular housing as a housing solution in urban areas a concept inseparable from the issue of housing affordability, which remains a structural challenge in Indonesia ( Perdamaian & Zhai, 2024; Helble et al., 2021).

Furthermore, this study explicitly considers differences in urban contexts as variables that may moderate the relative importance of each housing attribute. Different urban characteristics including economic structure, population density, purchasing power, infrastructure accessibility, and real estate market conditions shape distinct evaluation patterns and preference hierarchies among different social groups ( Analisa & Okada, 2023). Therefore, this research framework was developed not only to identify the housing attributes that most influence the acceptance of modular housing in general but also to compare the relative contributions of each attribute across various urban typologies in Indonesia. Through this cross-city comparative approach, the study aims to uncover variations in housing preference priorities shaped by differences in urban contexts, thereby yielding a more nuanced and contextual understanding of the determinants of modular housing acceptance in Indonesia. The conceptual framework underlying this study, which maps seven housing attribute constructs and their hypothesized effects on the level of acceptance of modular housing across various urban typologies, is illustrated in Figure 1.

**Figure 1.**
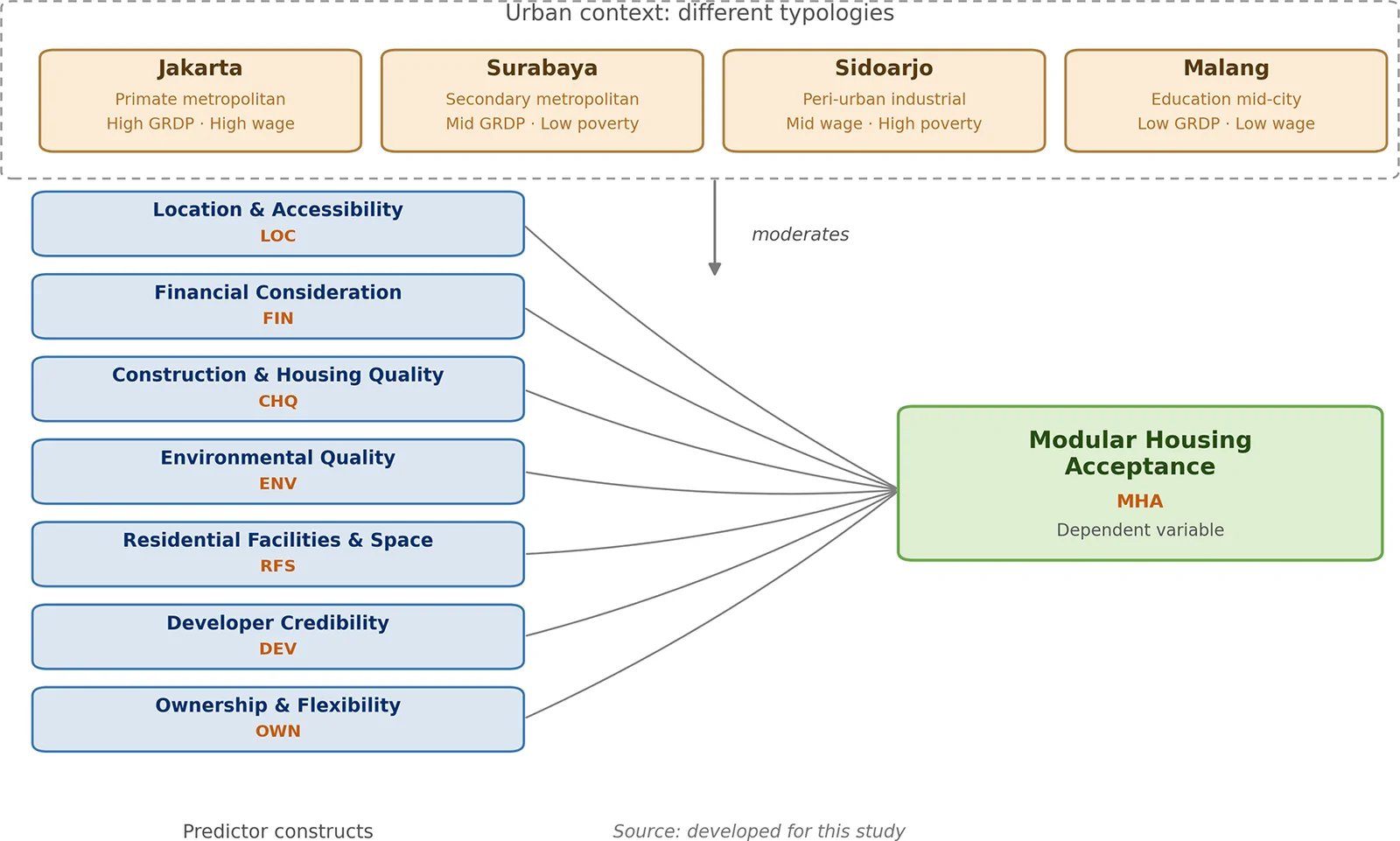
Theoretical framework for the acceptance of modular housing, showing the seven housing attribute constructs (location & accessibility, financial considerations, construction & housing quality, environmental quality, residential facilities & space, developer credibility, and ownership & flexibility) and their hypothesized influence on public acceptance of modular housing across different urban typologies.

As depicted in Figure 1, the framework positions the seven housing preference constructs (Location & Accessibility (LOC), Financial Considerations (FIN), Construction & Housing Quality (CHQ), Environmental Quality (ENV), Residential Facilities & Space (RFS), Developer Credibility (DEV), and Ownership & Flexibility (OWN)) as exogenous predictors with hypothesized direct paths to Public Acceptance of Modular Housing (MHA), the single endogenous construct. Each path represents an independent, non-overlapping contribution to MHA, consistent with the variance-decomposition logic underlying RWA. Urban typology is not modeled as an additional exogenous predictor but as a contextual moderator: it is hypothesized to leave the seven direct paths intact while reshaping their relative strength across metropolitan (Jakarta, Surabaya), peri-urban industrial (Sidoarjo), and education-oriented medium-sized (Malang) city contexts. This moderating role is operationalized empirically in the Methods through separate RWA estimation for each city, and its results motivate the typology-preference alignment concept developed in the Discussion.

### Research gap and contribution

Research on modular housing has grown rapidly in recent years, but most studies still focus on technical aspects, such as construction efficiency, productivity, sustainability, waste reduction, and building performance throughout its life cycle ( Saad et al., 2025; Zohourian et al., 2025). Meanwhile, research examining public acceptance of modular housing is relatively limited and generally focuses on perceptions of quality, economic benefits, or general acceptance of the technology ( Lee et al., 2019; Brickell et al., 2023). Consequently, there remains a limited understanding of how public preferences regarding housing attributes influence acceptance of modular housing.

This research gap becomes even more apparent in the context of developing countries, particularly Indonesia, which has highly diverse urban characteristics. Some studies in Indonesia address public housing preferences, while others focus on the potential for implementing modular construction. However, these two themes are generally still examined separately ( Analisa & Okada, 2023; Carissa et al., 2025). Furthermore, there is still very little research comparing the factors influencing the acceptance of modular housing across different urban typologies, even though differences in urban characteristics have the potential to result in different housing preferences.

Based on these gaps, this study makes three main contributions. First, this study expands the literature on modular housing acceptance by integrating the perspective of urban housing preferences as a foundation for understanding public acceptance of modular housing. Second, this study provides empirical evidence from Indonesia, a developing country facing complex challenges related to urbanization and housing provision. Third, this study reveals how housing attributes influence different urban typologies, thereby yielding a more contextual understanding of modular housing acceptance. These contributions are expected to support the development of housing acceptance theory while providing practical insights for governments and developers in designing modular housing implementation strategies that are better suited to the characteristics of each city.

## Methods

### Research design

This study employs a quantitative approach with a cross-sectional design to analyze the factors influencing public acceptance of modular housing in various types of cities in Indonesia. The quantitative approach was chosen because it allows for the objective and measurable testing of relationships between variables through standardized instruments, as is commonly applied in studies on housing preferences and the acceptance of innovations in the construction sector ( Yanti et al., 2020; Oluwunmi & Coker, 2024). Meanwhile, the cross-sectional design allows for data collection at a single point in time, thereby providing an overview of public preferences regarding modular housing and enabling comparisons across regions with different urban characteristics ( Yaacob et al., 2025; Rahman et al., 2024). Primary data were collected through a survey using a structured questionnaire distributed to respondents in four cities representing different urban characteristics: Jakarta, Surabaya, Sidoarjo, and Malang in 2025.

This study uses seven housing preference constructs as predictor variables and Public Acceptance of Modular Housing (MHA) as the dependent variable. These seven constructs include Location and Accessibility (LOC), Financial Considerations (FIN), Construction and Housing Quality (CHQ), Environmental Quality (ENV), Residential Facilities and Space (RFS), Developer Credibility (DEV), and Ownership and Flexibility (OWN). These constructs were developed based on the literature on housing preferences and the acceptance of modular housing, which indicates that people’s decisions in choosing housing are influenced by a combination of factors including location, financial considerations, building quality, the environment, facilities, and housing ownership characteristics ( Yanti et al., 2020; Adianto et al., 2023; Mangkunegara et al., 2021). The dependent variable MHA is measured through three indicators: homeownership preference, affordable purchase intention, and mortgage adoption intention. These three indicators represent the public’s level of acceptance of modular housing, ranging from ownership preferences to readiness to make a financial commitment to such housing products ( Yaacob et al., 2025). All items were measured using a five-point Likert scale, ranging from 1 (strongly disagree) to 5 (strongly agree).

Data analysis was conducted in two stages. The first stage involved evaluating the measurement model using Confirmatory Factor Analysis (CFA) to test construct validity and reliability. Convergent validity was assessed through factor loadings and Average Variance Extracted (AVE), while construct reliability was evaluated using Composite Reliability (CR). In addition, model fit was evaluated using several goodness-of-fit indices, namely the Comparative Fit Index (CFI), the Tucker–Lewis Index (TLI), the Root Mean Square Error of Approximation (RMSEA), and the Standardized Root Mean Square Residual (SRMR). CFA testing was conducted for all respondents without distinguishing between cities due to the limited number of respondents available for CFA analysis in each city.

The second stage employed Relative Weight Analysis (RWA) to identify the relative contributions of each housing preference construct to the acceptance of modular housing. This method was chosen because it can estimate the relative importance of each predictor despite correlations among independent variables a condition commonly encountered in research on consumer preferences and housing choice behavior ( Johnson, 2000; Tonidandel & LeBreton, 2011). The RWA results are expressed as relative weight percentages that sum to 100%, thereby enabling a clearer comparison of the factors that most strongly determine the acceptance of modular housing in each urban typology. Through this approach, the study not only identifies the dominant factors influencing the acceptance of modular housing but also reveals variations in public preference priorities across different urban contexts in Indonesia.

### Study area

This study was conducted in four cities in Indonesia that were purposively selected because they represent different typologies of urban development in Jakarta, Surabaya, Sidoarjo, and Malang. This diversity of typologies allows for a more comprehensive analysis of variations in public preferences for modular housing based on the urban context of each region. Differences in the levels of urbanization, population density, economic activity, and housing sector dynamics in each city are expected to influence residents’ needs and preference hierarchies when choosing housing ( Helble et al., 2021; Perdamaian & Zhai, 2024). The typologies of the four cities included in the study are summarized in Table 1 below.

**Table 1.** City typologies.

City	Urban typology	Province	Key characteristic
Jakarta	Primate metropolitan	DKI Jakarta	High density, high land price, national economic center
Surabaya	Secondary metropolitan	East Java	Industrial and trade city with high housing growth
Malang	Medium-sized city	East Java	Education and tourism city with gradual settlement development
Sidoarjo	Peri-urban	East Java	Surabaya’s buffer zone with rapid urbanization and housing development

Jakarta was selected as a representative of a primary metropolitan city (primate city) with a very high level of urbanization, high population density, and land prices that are relatively high by national standards, making it an area facing serious challenges in providing affordable and efficient housing ( Firmansyah et al., 2024). Surabaya was selected as a representative of a secondary metropolitan area characterized by rapid economic growth and housing development, yet with urban development characteristics distinct from Jakarta’s, including a more diverse real estate market structure and more moderate land-use pressures ( Santoso et al., 2019). Malang represents a medium-sized city typology centered on education and tourism that is experiencing gradual residential area development with lower population density, while Sidoarjo represents a rapidly developing peri-urban area due to the expansion of the Surabaya metropolitan area ( Firmansyah et al., 2024; Santoso et al., 2019).

To empirically strengthen the rationale for selecting these four cities, Table 2 presents the socioeconomic profiles of each city, including population size, GRDP per capita, minimum wage, and poverty rate ( BPS, 2025).

**Table 2.** City characteristics.

City	Population (Million Persons)	GRDP per capita (IDR Million/Year)	Minimum wage (IDR Million/Month)	Poverty rate (%)
Jakarta	10.68	344.35	5.40	4.28
Surabaya	3.02	283.31	4.96	3.56
Sidoarjo	2.02	145.53	4.87	4.40
Malang	0.88	122.92	3.51	3.85

Table 2 confirms the existence of substantial socioeconomic disparities among the four cities. Jakarta has the highest per capita GRDP (IDR 344.35 million/year) and the largest population (10.68 million people), while Malang has the lowest per capita GRDP (IDR 122.92 million/year) and the smallest population (0.88 million people). These differences in economic capacity, which are also reflected in the range of minimum wages from IDR 3.51 million/month (Malang) to IDR 5.40 million/month (Jakarta) are theoretically relevant because a city’s socioeconomic conditions directly shape its residents’ purchasing power, preferences, and housing acceptance patterns ( Helble et al., 2021). Previous studies have shown that high urban density correlates with increasing pressure on housing affordability and quality of life, making innovative construction solutions such as modular housing increasingly relevant in densely populated urban areas ( Kamali et al., 2025). Together, these four cities form a comprehensive spectrum of urban contexts ranging from primary metropolitan areas to peri-urban zones enabling an analysis of how residents’ preference hierarchies for modular housing vary according to the typology and socioeconomic capacity of each region ( Rahman et al., 2024).

### Population and sample

The study population consisted of adults (aged ≥18 years) residing in permanent housing in each of the cities. The sampling technique used was a combination of purposive sampling and quota sampling. Purposive sampling was used to ensure that each respondent met the established inclusion criteria, namely: (1) residing in one of the four study cities, (2) having experience in the process of selecting or occupying a residence, and (3) being willing to provide consent to participate. This approach is commonly used in housing preference studies that require respondents’ involvement in housing decision-making processes ( Yanti et al., 2020; Oluwunmi & Coker, 2024). Quota sampling was then applied to ensure a proportional and equitable distribution across cities, thereby enabling valid comparative analysis without regional representation bias. The minimum sample size was set at 300 respondents, based on the recommendation that multivariate analysis requires at least 200 respondents or at least 10 times the number of variables being analyzed ( Siddiqui, 2013).

A total of 300 respondents were successfully recruited, with an even distribution of 75 respondents per city. This balanced distribution ensures equal regional representation and enables meaningful cross-city comparative analysis. All respondents completed a structured questionnaire distributed offline, with inclusion criteria strictly applied at every stage of data collection. The final sample demonstrated diversity in demographic and socioeconomic characteristics, including age, gender, educational level, housing tenure status, and type of residence. This diversity enables a more robust and representative analysis of acceptance patterns of modular housing across urban contexts, while also providing an adequate empirical basis for linking acceptance findings to the specific socioeconomic profiles of each city, as detailed in subsection 3.2.

### Identification of research variables

Research variables were identified by selecting attributes from previously published works. Studies on housing preferences were thoroughly analyzed and synthesized to produce attributes that comprehensively reflect societal conditions. We identified various factors related to this topic within the existing literature. However, some attributes overlapped due to their similar characteristics. Therefore, we eliminated or combined these attributes into more meaningful and focused categories. In the final stage, we successfully identified 7 key attributes that describe housing preferences. In the final stage, we successfully identified seven main attributes that describe housing preferences, as shown in Table 3.

**Table 3.** Review of modular housing acceptance.

Variable	Indicator	Variable	Indicator
Location & Accessibility	Close to the city center	Environmental Quality	Clean and well-maintained environment
	Near public facilities		Adequate environmental safety
	Close to public transportation routes		Availability of a sufficient parking area
	Close to the main road		An environment that has privacy
	Far from traffic jams		Access to clean water channels
Financial Consideration	Installments that suit your financial capabilities	Construction & Housing Quality	Use of energy-efficient facade elements
	Low down payment		Durable material
	Flexible payment schemes		Strong building
	Selling price increases		Attractive final look
	Low maintenance costs		Quality materials
Ownership & Flexibility	Allows me to organize my home as I wish	Developer Credibility	Designed by professional architects
	Become an important asset and long-term investment		Built by a contractor with a trusted reputation
	I own land and buildings altogether		State-owned company contractor
	Accommodating space development		Extended building warranty
	Accommodate additional floors in multi-story buildings		Ready-to-occupy units
Residential Facilities & Space	Private garden	Public Acceptance of Modular Housing	Homeownership Preference
	Comfortable work space		Affordable Purchase Intention
	Warehouse/storage space		Mortgage Adoption Intention
	Spacious bedroom		
	Spacious bathroom		

These attributes were then grouped into eight main categories as shown in Table 3. These main categories include: Location & Accessibility, Financial Consideration, Construction & Housing Quality, Environmental Quality, Residential Facilities & Space, Developer Credibility, Ownership & Flexibility and Public Acceptance of Modular Housing. This results in a total of 38 relevant indicators (see Table 6 for the complete list of indicators and their factor loadings; the full questionnaire is available as extended data). To test the validity of these attributes, we conducted a series of interviews with experts in the construction and housing sectors. Furthermore, a questionnaire pilot test was conducted through several practical steps. With this approach, the research results can make a significant contribution to understanding community preferences in the context of modular housing development.

After identifying and mapping various factors potentially influencing public acceptance of modular housing, the next step in this research was to conduct further statistical tests. This stage is essential for confirming and measuring the significance of the relationships between the identified variables. Through in-depth statistical analysis, strong empirical evidence can be obtained regarding how each factor contributes to the adoption rate of modular housing.

### Informed consent

Prior to data collection, all prospective participants received an information sheet explaining the purpose of the study, the voluntary nature of participation, the confidentiality of their responses, and the fact that the data would be used solely for academic research purposes; this information sheet was included at the beginning of the questionnaire and listed the researcher’s contact details for further inquiries. Informed consent to participate was obtained from all participants prior to data collection. Written consent was obtained from participants who signed the consent form, while verbal consent was obtained by the researchers from participants who chose not to provide a written signature, as some respondents preferred to remain anonymous, were reluctant to sign a formal document, and the survey was conducted directly in the field. All responses were kept confidential and used solely for academic purposes.

### Data analysis

The collected data were analyzed in stages using two main methods: Confirmatory Factor Analysis (CFA) and Relative Weight Analysis (RWA). All analyses were conducted using R software version 4.3, with the lavaan package for CFA and the relaimpo package for RWA. This approach ensures that the research instruments have been validated before being used in the analysis of relative importance among constructs ( Tonidandel & LeBreton, 2011; Johnson, 2000).

CFA was applied to evaluate the validity and reliability of the measurement instruments based on a conceptual model developed from a review of the literature. CFA was used to ensure that each indicator accurately represents the latent construct being measured. Convergent validity was evaluated using two criteria: (1) factor loadings for each indicator ≥0.70, and (2) Average Variance Extracted (AVE) ≥ 0.50 at the construct level. The reliability of the instrument was evaluated using Composite Reliability (CR), with a value of ≥0.70 as the threshold. The overall model fit was assessed through a series of Goodness of Fit (GOF) indices, including a Comparative Fit Index (CFI) ≥ 0.95, a Tucker-Lewis Index (TLI) ≥ 0.95, Root Mean Square Error of Approximation (RMSEA) ≤ 0.08, and Standardized Root Mean Square Residual (SRMR) ≤ 0.08 ( Hair et al., 2019; Junianto et al., 2026). CFA was conducted on the entire sample (n = 300) to estimate the factor structure, factor loadings, AVE, and CR; subsequently, the GOF was evaluated separately for each city to verify the consistency of the factor structure across urban contexts.

After all constructs were psychometrically verified, the factor scores obtained from the CFA were used as inputs in the RWA. RWA was chosen to identify the relative contributions of each predictor construct to MHA due to its ability to address the issue of multicollinearity among predictors ( Takabatake & Hasegawa, 2022). Unlike standardized regression coefficients, which can be distorted by correlations among independent variables, RWA estimates the unique contribution of each predictor to the coefficient of determination (R ^2^) by decomposing the explained variance into relative weights that sum to 100% ( Tonidandel & LeBreton, 2011). The analysis results are presented in the form of relative weights and relative contribution percentages, followed by the ranking of the factors that most influence the acceptance of modular housing. The RWA was conducted separately for each study city to compare variations in the hierarchy of public preferences based on the urban typology of each region.

## Results

### Respondent characteristics

This section presents the demographic and socioeconomic profiles of the 300 respondents in this study, who were proportionally distributed across four urban and peri-urban areas in Jakarta, Surabaya, Sidoarjo, and Malang. Quota sampling was used to ensure adequate representation from each study location, with each city allocated a quota of 75 respondents. These four cities were selected based on the consideration that they represent diverse settlements ranging from large-scale metropolitan centers (Jakarta and Surabaya) to industry-based peri-urban areas (Sidoarjo) and medium-sized cities centered on education and services (Malang), thereby enabling a more comprehensive analysis of the acceptance of modular housing across different urban contexts. Respondent characteristics are presented in two separate tables: Table 4 presents the profiles of respondents in Jakarta and Surabaya, while Table 5 presents the profiles of respondents in Sidoarjo and Malang.

**Table 4.** Respondent characteristics Jakarta vs Surabaya.

Characteristic	Category	Jakarta		Surabaya	
		Freq	%	Freq	%
**Gender**	Male	38	50.67	44	58.67
	Female	37	49.33	31	41.33
**Age**	< 25 years	15	20.00	17	22.67
	25–34 years	44	58.67	28	37.33
	35–44 years	8	10.67	15	20.00
	45–54 years	6	8.00	9	12.00
	≥ 55 years	2	2.67	6	8.00
**Marital Status**	Married	44	58.67	29	38.67
	Unmarried	31	41.33	46	61.33
**Education**	Postgraduate	8	10.67	3	4.00
	Bachelor’s degree	58	77.33	26	34.67
	Diploma/Associate degree	0	0.00	3	4.00
	Senior high school	9	12.00	39	52.00
	Junior high school	0	0.00	4	5.33
**Housing Tenure**	Renting/Boarding	31	41.33	21	28.00
	Employer/Relative/Friend’s dwelling	3	4.00	2	2.67
	Owner-occupied	10	13.33	27	36.00
	Parental home	31	41.33	25	33.33
**Dwelling Type**	Apartment	3	4.00	4	5.33
	Four-storey boarding house	1	1.33	0	0.00
	Public housing/Walk-up flats	3	4.00	0	0.00
	Landed house (1–2 storeys)	68	90.67	71	94.67

**Table 5.** Respondent characteristics sidoarjo vs malang.

Characteristic	Category	Sidoarjo		Malang	
		Freq	%	Freq	%
**Gender**	Male	40	53.33	35	46.67
	Female	35	46.67	40	53.33
**Age**	< 25 years	11	14.67	8	10.67
	25–34 years	31	41.33	48	64.00
	35–44 years	20	26.67	10	13.33
	45–54 years	11	14.67	5	6.67
	≥ 55 years	2	2.67	4	5.33
**Marital Status**	Married	25	33.33	44	58.67
	Unmarried	50	66.67	31	41.33
**Education**	Postgraduate	2	2.67	10	13.33
	Bachelor’s degree	41	54.67	42	56.00
	Diploma/Associate degree	2	2.67	5	6.67
	Senior high school	30	40.00	18	24.00
	Junior high school	0	0.00	0	0.00
**Housing Tenure**	Renting/Boarding	17	22.67	8	10.67
	Employer/Relative/Friend’s dwelling	2	2.67	3	4.00
	Owner-occupied	24	32.00	19	25.33
	Parental home	32	42.67	45	60.00
**Dwelling Type**	Apartment	0	0.00	1	1.33
	Four-storey boarding house	0	0.00	0	0.00
	Public housing/Walk-up flats	0	0.00	0	0.00
	Landed house (1–2 storeys)	75	100.00	74	98.67

Table 4 shows that the gender distribution in Jakarta is relatively balanced between men (50.67%) and women (49.33%), while Surabaya is dominated by male respondents (58.67%). In terms of age, both cities are dominated by the 25–34 age group, but with significantly different proportions: Jakarta recorded 58.67%, while Surabaya recorded only 37.33%, with a more even distribution across older age groups. This dominance of the young working-age group is consistent with the findings of Mangkunegara et al. (2021), who identified that the segment of first-time homebuyers in that age range demonstrates greater openness to alternative and innovative housing solutions. Regarding marital status, Jakarta was dominated by married respondents (58.67%), whereas Surabaya was the opposite, with the majority of respondents unmarried (61.33%), reflecting the differences in the dominant life stages in the two cities.

Educational profiles show a fairly stark contrast: Jakarta is dominated by bachelor’s degree holders (77.33%) with an additional 10.67% holding master’s degrees, while Surabaya is dominated by high school graduates (52.00%) and only 34.67% hold bachelor’s degrees. This disparity has important implications in the context of acceptance of modular housing, given that educational level is positively correlated with technological readiness and perceptions of the benefits of construction innovations ( Shin et al., 2022), indicating that strategies for promoting modular housing need to be adapted to the local context. In terms of housing tenure status, Jakarta exhibits a distinctive duality: the proportions of renters and those living in their parents’ homes are both high (41.33% each), while homeownership stands at only 13.33%. This situation reflects the high pressure on property prices in DKI Jakarta, which theoretically creates latent demand for more affordable housing solutions ( Helble et al., 2021). Surabaya exhibits a more diverse distribution, with a higher proportion of owner-occupied housing (36.00%). Finally, the dominant housing type in both cities consists of one- to two-story single-family homes 90.67% in Jakarta and 94.67% in Surabaya reflecting Indonesians’ historical preference for landed property, which is associated with land ownership as a tangible asset and a marker of social status ( Akbar et al., 2023).

Table 5 shows that the gender distribution in Sidoarjo is slightly skewed toward males (53.33%), while Malang is dominated by females (53.33%). This variation aligns with the argument by Adianto et al. (2023) that housing preferences are also influenced by the dynamics of decision-making within households, where the composition and roles of family members shape considerations regarding the functionality and adaptability of space. In terms of age, Malang exhibits a very high concentration of the 25–34 age group (64.00%) the highest among the four cities which can be attributed to the large population of college students and young workers who remain in the city after completing their studies. Sidoarjo exhibits a more even age distribution, including a proportion of the 35–44 age group (26.67%), indicating that this peri-urban area is predominantly inhabited by families in the asset-consolidation phase. Marital status in Sidoarjo is dominated by unmarried respondents (66.67%) the highest proportion among the four cities while Malang features a majority of married respondents (58.67%), a pattern consistent with the age composition and social context of each region.

The educational profile in Sidoarjo shows a two-polar distribution between bachelor’s degree holders (54.67%) and high school graduates (40.00%), while Malang has the most balanced educational composition among non-metropolitan cities, with 56.00% holding bachelor’s degrees, 13.33% holding master’s degrees, and 6.67% holding associate’s degrees. The absence of respondents with a junior high school education background in both cities indicates that the sampled population possesses sufficient literacy skills to understand and evaluate the concept of modular housing. In terms of housing tenure status, Malang showed the highest proportion of respondents living in their parents’ homes (60.00%), a phenomenon known in housing literature as extended family living and representing an adaptive response to high property prices relative to income in the Indonesian urban context ( Adianto et al., 2023). Sidoarjo has a relatively high proportion of homeownership (32.00%), consistent with its character as an industry-based residential area with relatively stable purchasing power. Housing types in both cities consist almost entirely of single-family homes, with Sidoarjo recording 100% and Malang 98.67%, confirming the dominance of a preference for landed property a trend also observed in Jakarta and Surabaya though with a higher degree of homogeneity.

A cross-city comparison reveals several structural patterns that are important to consider in the analysis of modular housing acceptance. First, there is a clear gradient in educational profiles from Jakarta (highest) to Surabaya (relatively lower), with Malang and Sidoarjo occupying an intermediate position but with distinct characteristics, implying that perceptions of housing innovations vary across cities in line with differences in literacy levels ( Razkenari et al., 2020). Second, the dominance of the 25–34 age group across all cities peaking in Malang (64.00%) confirms that this study successfully reached the most relevant demographic segment as the target market for modular housing: millennials who are actively seeking their first housing solution ( Mangkunegara et al., 2021). Third, the high proportion of respondents who do not yet own independent housing whether renting or living with their parents found consistently across all four cities reflects a structural housing affordability crisis in Indonesia’s urban areas ( Helble et al., 2021), while also creating a socioeconomic context conducive to exploring housing alternatives such as modular systems. Fourth, the high prevalence of single-family homes across all study areas indicates that respondents’ frame of reference for evaluating modular housing is likely shaped by their experiences with and expectations of single-family homes, a factor that must be taken into account when interpreting acceptance variables in the subsequent analysis.

### Confirmatory factor analysis

To test the validity and reliability of the measurement instruments, this study applied Confirmatory Factor Analysis (CFA) to all constructs used. The evaluation was conducted using three main criteria: factor loadings as an indicator of item convergent validity, Average Variance Extracted (AVE) as a measure of construct convergent validity, and Composite Reliability (CR) as a measure of internal consistency. Referring to the thresholds established by Hair et al. (2019), an indicator is considered valid if it has a factor loading of ≥0.70; it is considered to have convergent validity if the AVE value is ≥0.50; and it is considered reliable if the CR value is ≥0.70. The complete CFA results are presented in Table 6 below.

**Table 6.** CFA results.

Variable	Indicator	Loading Factor	CR	AVE
**Location & Accessibility**	Close to the city center	0.812	0.908	0.664
	Near public facilities	0.847		
	Close to the main road	0.853		
	Close to public transportation routes	0.796		
	Far from traffic jams	0.784		
**Financial Consideration**	Installments that suit your financial capabilities	0.826	0.912	0.675
	Low down payment	0.847		
	Flexible payment schemes	0.793		
	Selling price increases	0.821		
	Low maintenance costs	0.839		
**Construction & Housing Quality**	Quality materials	0.853	0.925	0.711
	Durable material	0.828		
	Strong building	0.882		
	Attractive final look	0.846		
	Use of energy-efficient facade elements	0.811		
**Environmental Quality**	Clean and well-maintained environment	0.824	0.903	0.652
	Adequate environmental safety	0.836		
	Availability of sufficient parking area	0.782		
	An environment that has privacy	0.801		
	Access to clean water channels	0.797		
**Residential Facilities & Space**	Private garden	0.846	0.921	0.701
	Comfortable work space	0.831		
	Warehouse/storage space	0.817		
	Spacious bedroom	0.858		
	Spacious bathroom	0.847		
**Developer Credibility**	Designed by professional architects	0.833	0.914	0.681
	Built by a contractor with a trusted reputation	0.817		
	State-owned company contractor	0.796		
	Extended building warranty	0.856		
	Ready-to-occupy units	0.824		
**Ownership & Flexibility**	Allows me to organize my home as I wish	0.851	0.927	0.718
	Important long-term investment asset	0.823		
	Own land and buildings altogether	0.874		
	Accommodating space development	0.829		
	Accommodate additional floors	0.846		
**Public Acceptance of Modular Housing**	Homeownership Preference	0.891	0.918	0.789
	Affordable Purchase Intention	0.903		
	Mortgage Adoption Intention	0.874		

The CFA results show that all indicators across all variables met the specified validity thresholds. The Location & Accessibility variable obtained a CR value of 0.908 and an AVE of 0.664, with factor loadings ranging from 0.784 (far from traffic jams) to 0.853 (close to the main road). The Financial Consideration variable performed well with a CR of 0.912 and an AVE of 0.675, where the Low down payment indicator had the highest loading (0.847) and Flexible payment schemes had the lowest but still met the threshold (0.793). The Construction & Housing Quality variable recorded the second-highest AVE value among the predictor variables, namely 0.711 with a CR of 0.925, supported by the Strong building indicator, which had the highest loading (0.882), indicating that the perception of a building’s structural strength is the most dominant indicator in shaping perceptions of modular housing quality in this study’s sample.

The Environmental Quality variable obtained a CR of 0.903 and an AVE of 0.652, with a relatively even range of loadings from 0.782 (Availability of sufficient parking area) to 0.836 (Adequate environmental safety), indicating that all environmental quality indicators contribute proportionally to the latent variable. The Residential Facilities & Space variable recorded a CR of 0.921 and an AVE of 0.701, with the Spacious bedroom indicator being the most dominant (loading = 0.858) a finding consistent with the preference for bedroom space as a priority element in housing evaluations within the Southeast Asian context ( Yanti et al., 2020). The Developer Credibility variable obtained a CR of 0.914 and an AVE of 0.681, with the Extended building warranty indicator having the highest loading (0.856), indicating that an extended post-construction warranty is the strongest signal of credibility for respondents in evaluating modular housing developers a relevant finding given the still-limited track record of modular developers in Indonesia.

The Ownership & Flexibility variable recorded the highest AVE value among all predictor variables, namely 0.718 with a CR of 0.927, with the Own land and buildings altogether indicator having the highest loading (0.874). This confirms the argument by Akbar et al. (2023) that the simultaneous ownership of land and buildings is a deeply rooted housing aspiration within the Indonesian sociocultural context, making ownership flexibility the strongest indicator in defining this variable. Furthermore, the dependent variable Public Acceptance of Modular Housing demonstrated the best psychometric performance among all variables, with a CR of 0.918 and an AVE of 0.789 values that substantially exceed the conservative threshold of 0.70. The three indicators Homeownership Preference (0.891), Affordable Purchase Intention (0.903), and Mortgage Adoption Intention (0.874) all have loadings above 0.87, indicating that these three acceptance indicators consistently and strongly represent the latent variable of acceptance of modular housing.

Aggregately, the AVE values for all variables range from 0.652 to 0.789, all of which exceed the minimum threshold of 0.50, thus fulfilling the criteria for convergent validity. The CR values for all variables range from 0.903 to 0.927, far exceeding the 0.70 threshold, which confirms the composite reliability of all measurement instruments ( Hair et al., 2019). The feasibility of the CFA model was further evaluated through separate Goodness of Fit (GOF) tests for each city to ensure that the same factor structure applies consistently across all urban contexts studied. The GOF results are presented in Table 7 below.

**Table 7.** Goodness of fit by city.

City	CFI	TLI	RMSEA	SRMR
Jakarta	0.954	0.947	0.046	0.041
Surabaya	0.972	0.968	0.031	0.028
Malang	0.946	0.939	0.052	0.047
Sidoarjo	0.968	0.962	0.035	0.032

Table 7 shows that the CFA model has an acceptable fit in all cities. The CFI and TLI values are close to or above the 0.95 threshold, with the best performance in Surabaya (CFI = 0.972; TLI = 0.968) and the lowest performance slightly below the threshold in Malang (CFI = 0.946; TLI = 0.939). The RMSEA values for all cities were well below the tolerance limit of 0.08, ranging from 0.031 (Surabaya) to 0.052 (Malang), indicating a low level of model approximation error. SRMR values ranging from 0.028 to 0.047 also all met the threshold of ≤0.08, confirming that the average residuals between the observed and model covariance matrices were small.

### Relative weight analysis (RWA)

Relative Weight Analysis (RWA) was applied to identify the relative contributions of each predictor variable to public acceptance of modular housing in the four cities on a comparative basis. RWA was chosen because of its ability to address multicollinearity among predictor variables while simultaneously producing relative importance weights that sum to 100%, thereby facilitating a comparison of the proportional contributions of each predictor variable ( Johnson, 2000; Tonidandel & LeBreton, 2011). To facilitate interpretation, we rounded the relative weights to the nearest whole percentage when reporting the results. The RWA results are presented in Table 8 and Figure 2.

**Table 8.** Relative weight analysis results by city (%).

Variable	Jakarta	Surabaya	Sidoarjo	Malang
Location & Accessibility (LOC)	22	18	15	8
Financial Consideration (FIN)	18	16	22	10
Construction & Housing Quality (CHQ)	28	20	18	30
Environmental Quality (ENV)	10	10	8	15
Residential Facilities & Space (RFS)	8	14	14	12
Developer Credibility (DEV)	4	8	7	10
Ownership & Flexibility (OWN)	10	14	16	15
**Total**	**100**	**100**	**100**	**100**
R ^2^	0.781	0.864	0.842	0.731
Adjusted R ^2^	0.752	0.846	0.821	0.696

**Figure 2.**
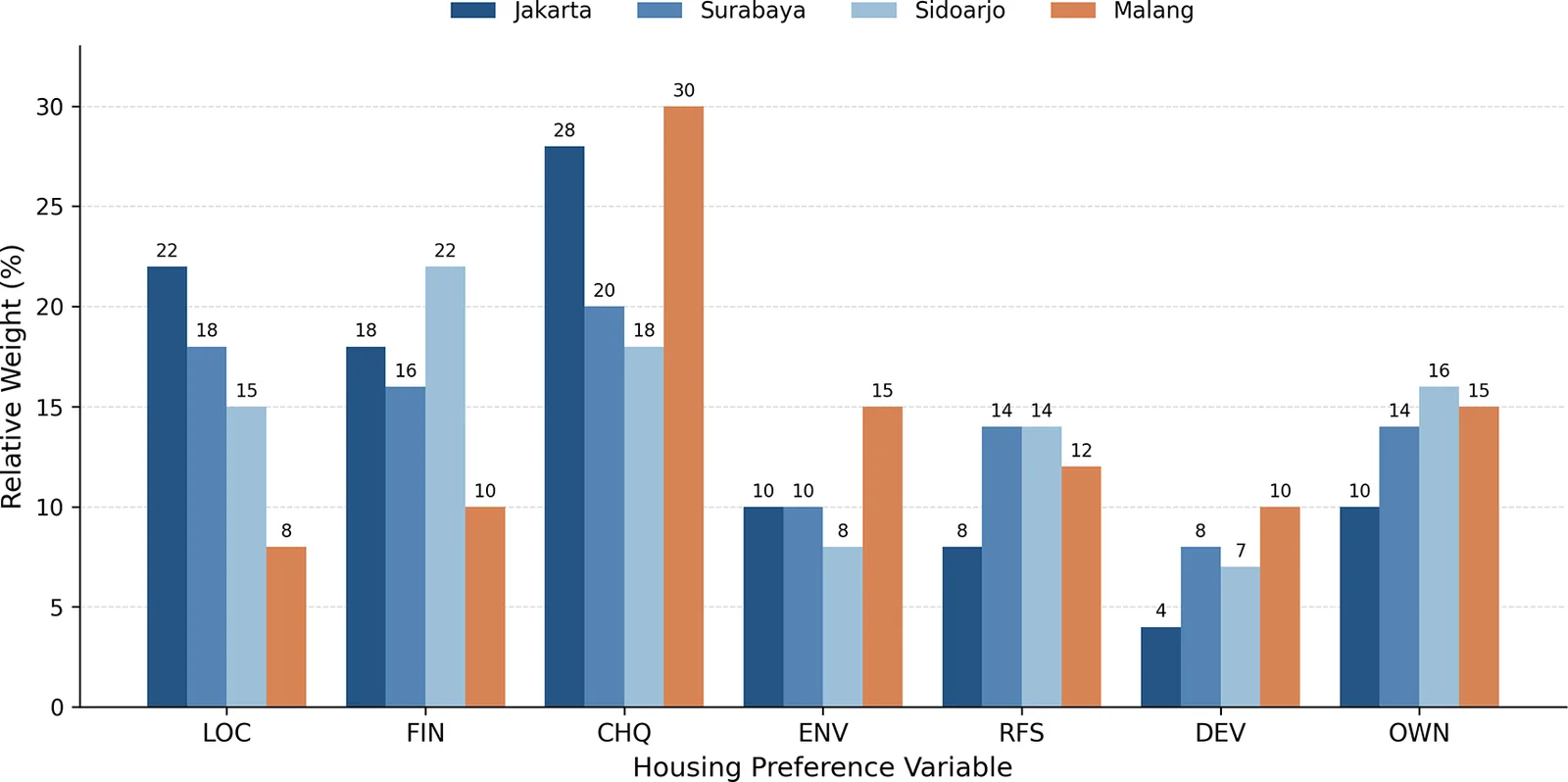
Relative weights (%) of the seven predictor constructs for public acceptance of modular housing by city (Jakarta, Surabaya, Sidoarjo, and Malang), derived from the relative weight analysis (RWA).

Table 8 shows that Construction & Housing Quality (CHQ) dominates the relative weight in three of the four cities, Jakarta (28%), Surabaya (20%), and Malang (30%) while Sidoarjo is the only city where Financial Consideration (FIN) ranks first (22%). On the other hand, Developer Credibility (DEV) consistently recorded the near-lowest weight across all cities, ranging from 4% to 10%, indicating a relatively stable hierarchy of importance despite significant contextual variations across regions. This weight distribution pattern is visualized in Figure 2 below to facilitate cross-city comparisons.

Across all four cities, the seven predictors collectively explained a substantial proportion of the variance in public acceptance of modular housing. The model fit was strongest in Surabaya (R ^2^ = 0.864, adjusted R ^2^ = 0.846) and Sidoarjo (R ^2^ = 0.842, adjusted R ^2^ = 0.821), followed by Jakarta (R ^2^ = 0.781, adjusted R ^2^ = 0.752) and Malang (R ^2^ = 0.731, adjusted R ^2^ = 0.696). The relatively small gap between R ^2^ and adjusted R ^2^ in each city indicates that the explanatory power is not inflated by the number of predictors, supporting the robustness of the models. Because the rescaled weights in Table 8 are proportional to these differing levels of model fit, they should be interpreted relative to the overall variance explained in each respective city.

Figure 2 reinforces the pattern evident in Table 8 while revealing nuances in the distribution that are not readily apparent from the numerical data alone. Visually, it is clear that CHQ forms the highest peak in almost all cities, with a striking gap compared to the other variables particularly in Malang, where the CHQ bar towers far above the other variables. In contrast, Sidoarjo appears the most visually distinct, with a prominent FIN bar and a more evenly distributed weighting among FIN, CHQ, and OWN. These visual variations across cities overall confirm that while CHQ is universally the most dominant factor, the relative weights of the other variables vary substantially according to each city’s socioeconomic context a finding that calls for a more in-depth city-by-city analysis, as outlined below.

The RWA results for Jakarta show that Construction & Housing Quality (CHQ) is the dominant predictor with the highest relative weight of 28%, followed by Location & Accessibility (LOC) at 22% and Financial Consideration (FIN) at 18%. Together, these three variables account for 68% of the total weight of importance, indicating that the acceptance of modular housing in Jakarta is largely determined by three key factors that are tangible and measurable. The dominance of CHQ in Jakarta can be understood in the context of high expectations regarding building quality in the nation’s business and economic hub, where respondents with higher education backgrounds tend to be more critical in evaluating the technical and aesthetic aspects of construction ( Razkenari et al., 2020). The high weight of LOC (22%) is also consistent with Jakarta’s characteristic as a city with chronic traffic congestion, where accessibility to the city center and public transportation are highly strategic considerations for housing ( Rajabi et al., 2024). Conversely, Developer Credibility (DEV) recorded the lowest weight in Jakarta (4%), likely because Jakarta respondents who are better educated and more exposed to information tend to have a higher capacity for independent evaluation and thus rely less on a developer’s reputation as an indicator of quality.

In Surabaya, the distribution of RWA weights is more even compared to other cities, with CHQ remaining in the top position (20%), though its margin of dominance is much smaller. LOC (18%), OWN (14%), and RFS (14%) follow with nearly equal weights, indicating that Surabaya respondents consider the acceptance of modular housing more holistically, without the dominance of any single extreme factor. This more balanced distribution pattern is consistent with Surabaya’s characteristics as Indonesia’s second-largest metropolitan city, which has relatively better-planned infrastructure compared to Jakarta, allowing various aspects of housing to be evaluated more proportionally. The higher OWN weight in Surabaya (14%) compared to Jakarta (10%) aligns with findings regarding respondent characteristics: Surabaya has the highest proportion of homeownership (36%), indicating that respondents who own or are in the process of owning a home are more sensitive to the flexibility and long-term investment value of modular housing ( Akbar et al., 2023).

The RWA results in Sidoarjo reveal a significantly different pattern, in which Financial Consideration (FIN) actually ranks first with a weight of 22%, surpassing CHQ (18%), which dominates in other cities. This finding has a strong contextual basis: as an industry-based peri-urban area, Sidoarjo has a population consisting largely of workers in the manufacturing and service sectors with income structures that are more vulnerable to fluctuations. Consequently, financial affordability considerations including installment plans, down payments, and flexible payment schemes become the primary determinants in housing decisions ( Rao & Biswas, 2023). Ownership & Flexibility (OWN) recorded a relatively high weight in Sidoarjo (16%) the highest among the four cities which can be attributed to the dominant proportion of unmarried respondents (66.67%) and those still living with their parents (42.67%), making housing flexibility to accommodate future needs a highly relevant consideration for this group. DEV again recorded a low weight (7%), consistent with patterns found in other cities.

Malang exhibits the most distinctive RWA profile among the four cities. CHQ recorded the highest overall weight across the entire dataset, at 30%, far surpassing the other variables. The very strong dominance of CHQ in Malang can be interpreted in the context of the city’s demographic composition, which is dominated by the 25–34 age group with a higher education background (postgraduate 13.33%; undergraduate degree 56%) and a majority who are married (58.67%) a profile that, in theory, correlates with higher expectations for long-term building quality, especially among those who view housing as a permanent family asset. The most notable aspect of Malang’s profile is the low weights for LOC (8%) and FIN (10%) both the lowest among the four cities indicating that respondents in Malang are relatively less sensitive to location and financial factors compared to respondents in other cities. This can be attributed to property prices in Malang being relatively more affordable than in Jakarta and Surabaya, so financial pressure is not a major obstacle, while the city’s more compact layout reduces the urgency of location considerations. Conversely, ENV (15%) and OWN (15%) received higher weights in Malang, indicating that environmental quality and ownership flexibility are significant secondary considerations after construction quality.

A comparison of the four cities revealed several important structural findings. First, CHQ consistently ranked in the top two across all cities, confirming that construction quality and materials are universal and context-independent factors in the acceptance of modular housing in Indonesia a finding consistent with the global literature on modular housing, which identifies perceptions of quality as both a barrier and a key driver in modular housing ( Kamali & Hewage, 2016). Second, there is a clear trade-off between LOC and FIN as the second-most dominant factors: cities with high accessibility pressures (Jakarta) prioritize LOC more highly, while cities with greater economic pressures (Sidoarjo) prioritize FIN more highly, indicating that the hierarchy of considerations for modular housing is context-specific and cannot be generalized uniformly. Third, DEV consistently recorded the lowest or near-lowest weight across all cities (Jakarta: 4%; Sidoarjo: 7%; Surabaya: 8%; Malang: 10%), which, while not interpretable as absolute unimportance, indicates that in the context of decisions regarding the acceptance of modular housing, intrinsic product factors (quality, location, financial aspects) dominate the evaluation more than extrinsic factors such as the developer’s reputation. This finding has relevant practical implications for modular housing developers in Indonesia, namely that investing in product quality and affordability will yield a greater impact on acceptance than investing solely in branding and institutional credibility.

## Discussion

The findings of this study provide a substantive empirical contribution to the understanding of the acceptance of modular housing in urban Indonesia, while also revealing dynamics that cannot be fully explained by theoretical frameworks developed in the context of developed countries. The following discussion integrates the RWA results with the typological profiles of each city which encompass both socioeconomic dimensions (GRDP per capita, minimum wage, poverty rate) and urban-functional dimensions (primary metropolitan areas, secondary metropolitan areas, industrial peri-urban areas, and medium-sized educational cities) to produce a contextual and multi-layered interpretation.

It should be noted that the demographic- and typology-based explanations offered throughout this discussion are narrative interpretations grounded in descriptive patterns and prior literature; they were not tested using formal statistical procedures, such as moderation or interaction analyses, within the present RWA framework. These interpretations should therefore be read as plausible, tentative explanations rather than statistically confirmed mechanisms, and are proposed primarily to guide future confirmatory research.

### Jakarta as primary metropolitan city with high expectations for quality and accessibility challenges

Jakarta, as the national economic and administrative center with the highest per capita GRDP (IDR 344.35 million/year) and a minimum wage of IDR 5.40 million/month, represents the typology of a high-income primate city in the Indonesian context. This profile is directly reflected in the RWA weighting structure, where Construction & Housing Quality (CHQ) dominates at 28% and Location & Accessibility (LOC) ranks second at 22%. The dominance of CHQ in Jakarta confirms the findings of Razkenari et al. (2020), who found that a highly educated urban population with stronger purchasing power tends to regard construction quality as the primary predictor of acceptance of housing innovations, as they have the capacity to compare and evaluate building technical standards more critically. This is reinforced by the educational profile of Jakarta respondents, which is dominated by bachelor’s degree holders (77.33%) and master’s degree holders (10.67%) the highest proportion among the four cities.

The high weight of LOC (22%) in Jakarta also confirms the argument by Rajabi et al. (2024) that spatial accessibility is a structural consideration for housing in cities with chronic traffic congestion and high dependence on daily mobility. This finding aligns with the study by Pan et al. (2023), which identified that in densely populated Asian cities with unevenly distributed transportation infrastructure, the proximity of housing to economic activity centers is a significant differentiating factor in property purchase decisions. One interesting finding that slightly diverges from the literature is the low weight of Financial Consideration (FIN) in Jakarta (18%), which is relatively lower than that of LOC, given that Jakarta actually has the highest property prices nationwide. This phenomenon can be explained by two complementary mechanisms: first, the relatively high income of Jakarta respondents reflected in the highest minimum wage and the largest per capita GRDP proportionally reduces price sensitivity; second, the equally high proportions of renters and those living with their parents (41.33% each) indicate that most respondents are not yet in the active home-buying phase, making short-term financial considerations less urgent than an evaluation of product quality.

### Surabaya as a secondary metropolitan city with a holistic distribution of preferences

Surabaya, as a secondary metropolitan area with a per capita GRDP of IDR 283.31 million per year and the lowest poverty rate among the four cities (3.56%), exhibits a different typological profile from Jakarta, even though both have metropolitan status. The RWA results consistently reflect this difference: the distribution of weights in Surabaya is much more even, with CHQ (20%), LOC (18%), OWN (14%), and RFS (14%) forming clusters of interest that lack any single extreme dominance. This pattern aligns with the findings of Sohaimi et al. (2025) in the context of Kuala Lumpur a city with a comparable typology who found that among the middle-class urban population with relatively stable welfare levels, housing evaluations tend to be multidimensional because no single factor acts as a dominant bottleneck in decision-making.

The higher weight of Ownership & Flexibility (OWN) in Surabaya (14%) compared to Jakarta (10%) is a theoretically interesting finding. Surabaya recorded the highest proportion of single-family home ownership among the four cities (36%), indicating that respondents in Surabaya are in a more financially mature stage of life and are more actively considering housing as a long-term investment vehicle. This is consistent with the argument by Akbar et al. (2023) that populations with prior property ownership experience tend to evaluate flexibility and potential for value appreciation as more critical housing attributes. This finding also confirms the relevance of the value proposition of modular housing particularly its capacity for vertical expansion and structural modifications for a market segment that is already economically established but seeks flexibility in adapting their housing as family needs change.

### Sidoarjo as a peri-urban industrial city with predominantly financial sensitivity

Sidoarjo, as an industry-based peri-urban area, exhibits socioeconomic characteristics that differ from those of Jakarta and Surabaya. With a per capita GRDP of IDR 145.53 million per year and the highest poverty rate among the four cities studied (4.40%), Sidoarjo represents a context that is more sensitive to economic considerations in housing decision-making. This situation is reflected in the results of the Relative Weight Analysis (RWA), which identifies Financial Consideration (FIN) as the most dominant predictor of acceptance of modular housing, with a relative weight of 22%. Unlike other cities that place greater emphasis on building quality, the people of Sidoarjo tend to evaluate modular housing based on financial affordability, including the ability to make monthly payments, the size of the down payment, and long-term cost efficiency. This finding supports the argument by Pan et al. (2023) that affordability is a key factor in the acceptance of construction innovations among communities with relatively limited economic capacity.

Interestingly, Ownership and Flexibility (OWN) also received a high weight (16%), making it the second most important factor after FIN. This finding indicates that the people of Sidoarjo not only consider their current ability to purchase a home but also prioritize the housing’s ability to adapt to future needs. The high preference for flexibility can be linked to the characteristics of the respondents, who are predominantly unmarried individuals still living with their parents; thus, housing is viewed as a long-term asset that must be able to accommodate changes in family needs and household economic development. In this context, flexibility is not perceived as a premium feature that increases costs, but rather as a strategy to mitigate the risks of future uncertainty. These findings expand the literature on the acceptance of modular housing by demonstrating that considerations of affordability and flexibility can operate simultaneously as key determinants, particularly in peri-urban areas undergoing economic and demographic transitions.

### Malang as a medium-sized city of education with a focus on absolute quality

Malang, a medium-sized city known as an educational hub in East Java, exhibits different preference patterns compared to other cities. Although it has the lowest GRDP per capita among the four cities studied (IDR 122.92 million per year), the results of the Relative Weight Analysis (RWA) show that Construction and Housing Quality (CHQ) is the most dominant factor with a relative weight of 30%, while Location and Accessibility (LOC) and Financial Considerations (FIN) contribute only 8% and 10%, respectively. These findings indicate that the acceptance of modular housing in Malang is determined more by perceptions of building quality than by considerations of location or financial affordability. Thus, the emerging pattern does not fully align with the assumption that regions with lower economic capacity would be more sensitive to price factors in housing decision-making.

This pattern can be understood through the demographic characteristics and urban functions of the City of Malang. Respondents in Malang are dominated by the young working-age group, have the highest proportion of postgraduate education, and the majority are married. These characteristics indicate the presence of young professionals and an educated population who tend to evaluate housing based on long-term benefits, including construction quality, building durability, and comfort of use. This finding is consistent with Analisa and Okada (2023), who show that groups with higher educational levels tend to prioritize quality and sustainability over price considerations alone. Furthermore, the low contribution of LOC can be explained by Malang’s relatively compact spatial characteristics, with lower traffic congestion levels compared to Jakarta and Surabaya; consequently, accessibility is not a primary concern in housing selection. These results indicate that the relationship between regional economic conditions and housing preferences is not linear but is influenced by demographic characteristics as well as the city’s own functions. Thus, the findings from Malang provide evidence that building quality can be a key determinant of the acceptance of modular housing even in areas with relatively lower levels of economic prosperity.

From a typological perspective, there are three patterns worth discussing within the broader context of the literature. First, the universality of CHQ as a dominant predictor across all typologies primary metropolitan, secondary metropolitan, industrial peri-urban, and medium-sized educational cities confirms the findings of Kamali & Hewage (2016), who identified perceptions of quality as the most context-transcending factor in the acceptance of modular housing. This finding has important implications: while marketing and communication strategies for modular housing need to be adapted to each city typology, investing in improved material and construction quality is a universal strategy relevant across all urban contexts in Indonesia.

Second, this study identifies a consistent typology-preference alignment across all cities, where cities with high accessibility pressures (Jakarta, a primary metropolitan area) prioritize LOC more highly; cities with greater economic pressure (Sidoarjo, an industrial peri-urban area) prioritize FIN more highly; cities with a dominant academic population (Malang, an educational city) prioritize CHQ more highly in absolute terms; and cities with relatively stable well-being (Surabaya, a secondary metropolitan area) exhibit a holistic distribution. This pattern confirms and expands upon the argument by Adianto et al. (2023) that housing preferences are context-specific, yet offers an additional contribution by demonstrating that urban typology rather than merely income level serves as a more predictive categorization framework for understanding the hierarchy of modular housing preferences.

Theoretically, the typology-preference alignment pattern observed in this study can be situated within the broader person-to-environment fit tradition in housing research. This tradition holds that residential satisfaction and acceptance stem from congruence between individual or household needs and the affordances of the surrounding urban environment, as shown by Adianto et al. (2023) and Analisa and Okada (2023). Framed in this way, urban typology functions as a proxy for a bundle of environmental affordances and constraints. These include population density, land price, economic base, and demographic composition, and they jointly shape which housing attributes become salient to residents. This positions typology-preference alignment as an extension of existing preference-context theory, applied specifically to the modular housing domain, rather than as a wholesale replacement of that theory. At the same time, because the pattern is derived inductively from only four cities without formal significance testing of city-level differences, as noted in the Limitations, it should be regarded as a conceptual heuristic that still requires confirmatory testing. Future confirmatory testing could involve, for example, cluster analysis of a larger set of cities using objective typology indicators such as population density, GRDP per capita, and land price, or moderated regression that treats urban typology as a formal moderator, before this pattern can be considered an established theoretical framework.

Third, the consistently low weight of Developer Credibility (DEV) across all city typologies (4%–10%) is a finding that partially contradicts the literature on construction innovation adoption, which generally positions institutional trust as a critical factor in the acceptance of new building technologies ( Saad et al., 2025). One possible explanation is that, in the Indonesian context, trust in developers is built more through informal reputation and social networks (word-of-mouth) than through formal attributes such as certifications or institutional track records a dynamic that warrants further exploration in future research. Another alternative explanation is that the low weight of the DEV factor reflects the early stage of modular housing adoption in Indonesia, where respondents do not yet have sufficient experience to distinguish the credibility of different modular developers, so their evaluations rely more heavily on product attributes that can be assessed directly.

### Implications

The findings of this study have several practical implications relevant to two key stakeholder groups in Indonesia’s modular housing ecosystem, namely, property developers and housing policymakers, both of whom need to consider the heterogeneity of urban contexts as a strategic variable, not merely a backdrop, when designing modular housing products and regulations.

For property developers, the finding that Construction & Housing Quality (CHQ) is a universal and dominant predictor of acceptance across all city typologies sends a clear signal that investing in material quality, structural strength, and the building’s final aesthetic is a strategy that yields the highest return on acceptance across markets. Developers marketing modular housing in Indonesia can no longer rely solely on the narrative of cost efficiency and construction speed value propositions that have long dominated modular housing marketing communications in global markets ( Saad et al., 2025) without simultaneously building a strong and verified perception of quality. However, product and communication strategies must be adapted contextually according to each city typology. In Jakarta, developers should emphasize the advantage of location accessibility as a complementary attribute to construction quality, given the high weighting of Location & Accessibility (LOC) in this market; a strategy of locating projects near public transportation corridors and business centers will serve as a significant differentiator. In Sidoarjo, flexible and affordable financing schemes, including low down payments, adaptive installment plans, and partnerships with microfinance institutions, are essential prerequisites, given that Financial Considerations (FIN) are the primary predictor in this peri-urban market. In Surabaya, developers need to position modular housing as a flexible long-term investment vehicle, highlighting its expandability and structural adaptability as added value for segments that already own or are in the process of acquiring property. In Malang, a communication strategy emphasizing technical superiority and material sustainability will be more effective than arguments based on affordability, given the absolute dominance of CHQ and low financial sensitivity in this educational city’s market. Across cities, the consistently low weight of Developer Credibility (DEV) indicates that investment in institutional branding needs to be balanced or even prioritized by investment in product quality that prospective residents can directly experience; show units, transparent post-construction guarantees, and testimonials from the local community are likely more effective in building trust than formal certifications alone.

For housing policymakers, these research findings underscore the need for regulatory and incentive approaches that are place-sensitive rather than uniformly applied nationwide. The predominance of financial considerations in Sidoarjo and similar peri-urban areas indicates that, if designed, modular housing subsidy programs would achieve the greatest impact if focused on peri-urban and industrial areas, where affordability constraints represent the most significant structural barriers. Local governments in these areas could consider incentive schemes such as tax breaks for modular developers offering prices below a certain threshold, or subsidized mortgage programs specifically designed for modular units. On the other hand, the finding that a large proportion of respondents across all cities still live with their parents or are renters reflects a significant but unmet latent demand, indicating that policies facilitating access to first-time buyer financing for modular housing have the potential to open up a market segment that has long been underserved by conventional housing schemes. From a land-use planning perspective, the consistently high preference for landed houses across all cities, ranging from 90.67% to 100%, implies that zoning regulations need to more explicitly accommodate the development of modular landed housing, including the streamlining of Building Construction Permit (IMB) procedures for modular construction systems, which currently remain in a regulatory gray area in many regions. The development of national standards (Indonesian National Standards/SNI) that specifically regulate the quality and safety of modular construction is also an urgent need, given that the universality of CHQ as a predictor of acceptance indicates that regulatory quality assurance will have a broad positive impact on public confidence in modular housing as a whole.

## Conclusion

This study aims to identify the factors that influence public acceptance of modular housing in four types of cities in Indonesia, namely Jakarta, Surabaya, Sidoarjo, and Malang. The results indicate that all constructs used meet the criteria for validity and reliability, making them suitable for explaining public preferences regarding modular housing. Key findings show that Construction & Housing Quality is the most consistent factor influencing the acceptance of modular housing in most cities, underscoring the importance of perceptions of building quality in driving the adoption of new construction technologies.

Relative Weight Analysis reveals that public preference priorities vary according to each city’s characteristics. Jakarta places greater emphasis on location and accessibility; Sidoarjo shows a dominance of financial considerations; Surabaya exhibits a relatively balanced pattern of preferences; while Malang is more oriented toward housing quality. These findings indicate that the acceptance of modular housing is not only determined by product characteristics but is also influenced by the socioeconomic context and urban characteristics of the communities where people live.

Theoretically, this study demonstrates that the relationship between housing preferences and the acceptance of modular housing is contextual and influenced by urban typology. We propose typology-preference alignment as an initial conceptual framework grounded in patterns observed across four Indonesian cities. This framework should be treated as a heuristic for guiding future, larger-sample confirmatory studies rather than as a statistically validated typology. Practically, the research results can serve as a basis for developers, urban planners, and policymakers in designing modular housing development strategies that are better suited to the needs of communities in areas with different urban characteristics.

This study has several limitations. First, the data were collected through perception-based questionnaires, so they may still contain subjective biases on the part of respondents. Second, the scope of the study was limited to four cities on the island of Java, so generalizing the results requires caution. Third, the cross-sectional research design was unable to capture changes in public preferences over time. Fourth, although the total sample (N = 300) was adequate for the pooled CFA, the per-city subsample (n = 75) is relatively small for conducting city-level CFA and goodness-of-fit evaluation. Model fit indices at this sample size can be less stable and more sensitive to minor specification differences, so the city-specific fit results reported in Table 7, and the corresponding RWA estimates, should be interpreted as indicative rather than definitive. Replication with larger city-level samples is recommended to confirm the stability of the measurement model across urban typologies. Therefore, future research is recommended to expand the study area to cities outside Java, employ a longitudinal approach, and consider additional variables such as technological literacy, experiences with modular housing, and the influence of digital media to gain a more comprehensive understanding of the acceptance of modular housing in Indonesia.

## Ethical considerations

This study is a non-invasive, low-risk social survey that does not involve medical procedures, physical interventions, or sensitive legal investigations. This study was conducted in accordance with the research ethics principles and guidelines of Brawijaya University, administered by the Research and Innovation Ethics Committee (KERIS UB; https://keris.ub.ac.id/guideline-etik/), as well as applicable national regulations regarding social science research involving human participants.

## Data Availability

OSF: Urban Housing Preferences and Public Acceptance of Modular Housing Across Different City Typologies in Indonesia. https://doi.org/10.17605/OSF.IO/GRQU7 ( Pratama R et al., 2026). This project contains the following underlying data:
- Dataset_anonymous.xlsx (Anonymised survey response data from 300 respondents across different city typologies in Indonesia, including household income categories, monthly expenditure, estimated mortgage repayment capacity, financial freedom level, and responses to Likert-scale items measuring housing preferences and acceptance of modular housing) OSF: Urban Housing Preferences and Public Acceptance of Modular Housing Across Different City Typologies in Indonesia. https://doi.org/10.17605/OSF.IO/GRQU7 ( Pratama R et al., 2026). This project contains the following extended data:
- Research questionnaire (English).docx (the structured questionnaire, comprising 61 five-point Likert-scale items across the seven housing attribute constructs, used for data collection) -
Informed_Consent_Form.docx (consent form regarding the explanation of the research provided to respondents) Data are available under the terms of the Creative Commons Zero “No rights reserved” data waiver (CC0 1.0 Public domain dedication). Articles in F1000Research must comply with consensus-based minimum reporting guidelines for research. Comprehensive lists of available reporting guidelines can be found on the EQUATOR network website for health research. This study followed the STROBE checklist for cross-sectional studies. The completed checklist is provided as a supplementary file.
