# Supplementary material for: Urban Housing Preferences and Public Acceptance of Modular Housing Across Different City Typologies in Indonesia
Source: F1000Res. 2026 Nov 3;15:1254. Originally published 2026 Jul 29. [Version 2] doi: 10.12688/f1000research.185553.2 (PMC13740456; doi:10.12688/f1000research.185553.2)
Supplement: Peer review history

# Reviewer report: Reviewer response for version 2

**DOI:** 10.5256/f1000research.213861.r512786

**Published:** 2026-11-19

**Authors:** Mohamed Aly Etman

**Affiliations:** 1 University at Buffalo, Buffalo, NY, USA

This manuscript examines the relationship between housing preferences and public acceptance of modular housing across four Indonesian cities: Jakarta, Surabaya, Sidoarjo, and Malang. The authors use survey responses from 300 participants, equally distributed across the four cities, and apply Confirmatory Factor Analysis (CFA) to evaluate seven housing preference constructs, followed by Relative Weight Analysis (RWA) to identify their relative contributions to modular housing acceptance.

 The findings suggest that Construction and Housing Quality is the highest-ranked predictor in Jakarta, Surabaya, and Malang, while Financial Considerations rank first in Sidoarjo. Based on these differences, the authors propose the concept of "typology-preference alignment" to explain how housing priorities may vary across different urban contexts.

 The topic is relevant to housing research, construction innovation, and urban planning, particularly given the growing interest in prefabricated and modular construction as an alternative approach to housing delivery. The comparative perspective is a strength, and the manuscript is generally well structured and supported by relevant literature. The revised version also provides a clearer theoretical framework and acknowledges several limitations identified during previous reviews.

 My main concern is not with the relevance of the research question, but with the extent to which the statistical analysis supports the interpretation of differences across urban typologies. The study provides useful exploratory evidence, but some of the conclusions and policy recommendations go beyond what can be established from the current analysis.

 Major comments: issues to be addressed

**1. Cross-city comparisons require stronger statistical support.**

 The central contribution of the study is the identification of different housing preference hierarchies across urban typologies. However, the results are based on separate RWA models for each city, with only 75 respondents per city. The ranking of predictors provides useful descriptive information, but it does not establish whether the differences between cities are statistically significant.

 For example, Construction and Housing Quality accounts for 28% of the relative weight in Jakarta and 30% in Malang. These values are interpreted as reflecting different urban contexts, but the manuscript does not provide a statistical basis for determining whether this difference is meaningful.

 The authors' response to the previous review indicates that bootstrap confidence intervals were added to Table 8. However, the revised table reports only percentage weights, R², and adjusted R². No confidence intervals are visible.

 This discrepancy should be resolved. The authors should report bootstrap confidence intervals for the relative weights and, where feasible, assess the uncertainty associated with differences between cities. At a minimum, the findings should be consistently described as descriptive differences rather than statistically established differences between urban typologies.

**2. The measurement model requires additional clarification.**

 The use of CFA to validate the housing preference constructs is appropriate in principle. The reported factor loadings, composite reliability, and average variance extracted values indicate satisfactory convergent validity.

 However, there is an important methodological question concerning cross-city measurement consistency. The authors report that the CFA was estimated using the pooled sample of 300 respondents, while goodness-of-fit indices were subsequently evaluated separately for each city. Given the relatively small city-level samples, the stability of these estimates needs to be considered carefully.

 More importantly, acceptable goodness-of-fit values across cities do not by themselves demonstrate measurement equivalence. Because the study compares the relative importance of latent constructs across different urban populations, the authors should explain whether measurement invariance was evaluated.

 A multi-group CFA assessing at least configural and metric invariance would strengthen the analysis. If the available sample does not permit a reliable assessment, the authors should acknowledge this limitation and avoid assuming that the constructs are measured equivalently across cities.

 The manuscript should also clarify how the CFA factor scores were calculated and subsequently used in the city-level RWA models.

**3. Urban typology is not independently tested as an explanatory variable.**

 The distinction between the four cities is central to the paper's theoretical contribution. However, each urban typology is represented by only one city. Consequently, the analysis cannot distinguish the effects of urban typology from other characteristics specific to each city.

 The respondent profiles also differ substantially across the four locations, particularly regarding age, education, marital status, and housing tenure. These demographic differences may explain some of the observed variation in housing preferences independently of urban typology.

 Although the authors acknowledge that their demographic explanations are interpretive rather than statistically tested, other sections continue to attribute differences directly to urban characteristics.

 The authors should more consistently distinguish between observed city-level differences and demonstrated effects of urban typology. If possible, a supplementary analysis controlling for key respondent characteristics would help determine whether some of the differences remain after accounting for sample composition.

 The proposed typology-preference alignment concept is potentially useful, but should remain an exploratory conceptual interpretation rather than a validated explanatory framework.

**4. The sampling strategy limits the generalizability of the results.**

 The combination of purposive and quota sampling is reasonable for an exploratory housing preference study. However, the manuscript describes the balanced allocation of 75 respondents per city as ensuring representative comparisons.

 Equal sample sizes improve the balance of the analysis, but they do not establish representativeness within each city. The respondents were not selected through probability sampling, and the demographic profiles indicate that some groups are substantially overrepresented.

 For example, 77.33% of the Jakarta respondents hold bachelor's degrees, and most respondents across the four cities occupy one- or two-storey landed houses. Such characteristics may shape perceptions of modular housing and limit the applicability of the results to broader housing markets.

 The authors should provide more information about respondent recruitment, survey locations, and quota criteria. They should also clarify the intended target population and moderate claims about the representativeness of the sample.

**5. Reproducibility of the statistical analysis needs improvement.**

 The manuscript provides the research constructs, the indicators, the main statistical methods, and a link to the underlying dataset. These are positive steps toward transparency.

 However, several details need clarification.

 First, the Data Availability section describes a questionnaire containing 61 Likert-scale items, while the Methods and CFA tables identify 38 measurement indicators. The authors should explain whether the additional items were demographic questions, screening questions, or indicators excluded during model development, and provide the criteria used for any exclusions.

 Second, the manuscript identifies the relaimpo package for RWA, but does not specify the particular algorithm or function used. This is important because relative importance methods can produce different variance allocations.

 Third, the analysis should be accompanied by reproducible R scripts, including the CFA specifications, factor-score extraction, RWA calculations, and uncertainty estimation.

 Providing these materials would allow other researchers to verify the results and extend the analysis to additional cities.

**6. Interpretation of relative weights and statistical results should be more precise.**

 RWA is useful for identifying the relative contribution of correlated predictors to explained variance, but it does not establish causal relationships or the direction of their effects.

 Throughout the manuscript, the results are frequently described in terms of factors that "determine," "drive," or "influence" acceptance. Given the cross-sectional observational design, these terms should be used cautiously. The authors should emphasize that the analysis identifies associations and relative contributions to the explained variance in acceptance.

 The manuscript also interprets lower relative weights as evidence of lower sensitivity to particular housing attributes. For example, the low Financial Considerations weight in Malang is used to suggest that affordability is less of an obstacle. However, relative weights are conditional on the other predictors in the model and should not be interpreted as direct measures of affordability, willingness to pay, or absolute importance.

 The authors should also verify the reported adjusted R² values against the specified regression models, as the values in Table 8 do not appear to follow the conventional adjustment for seven predictors and 75 observations per city.

 Additional comments

**7. Strengthen the distinction between modular housing acceptance and general housing preferences.**

 Several of the measured attributes, including accessibility, financing, environmental quality, and residential facilities, are relevant to housing selection generally rather than modular housing specifically.

 The authors should clarify how modular construction was introduced and explained to respondents before completing the questionnaire. This is particularly important where respondents may have limited prior knowledge of the technology.

 The three acceptance indicators also focus on homeownership, affordable purchase intention, and mortgage adoption. The authors should discuss whether these sufficiently capture acceptance of modular construction itself, as opposed to general housing purchase intentions.

**8. Connect the findings more directly to housing design and construction implications.**

 The paper would benefit from a stronger discussion of how the identified preferences relate to specific characteristics of modular housing.

 For example, Construction and Housing Quality includes material durability, structural strength, aesthetics, and energy-efficient facades. These represent different aspects of building performance and design quality. It would be useful to distinguish these dimensions when discussing implications for modular housing development.

 Similarly, Ownership and Flexibility could be connected more explicitly to the potential of modular systems for spatial adaptability, incremental expansion, and long-term housing modifications.

 These connections would make the findings more relevant to architects, housing designers, and construction researchers.

**9. Reconsider the strength of the policy recommendations.**

 The discussion includes recommendations concerning housing subsidies, financing incentives, zoning regulations, and construction standards. While these are relevant topics, they are not directly evaluated in the study.

 The authors should frame these recommendations as potential directions for future policy evaluation rather than as established interventions supported by the survey findings.

**10. Improve presentation and consistency.**

 The overall organization is clear, but several sections repeat the same city-level findings and their interpretations. The Discussion could be condensed to focus more on comparative insights and theoretical implications.

 The manuscript should also consistently distinguish between the reported relative weight percentages and the underlying model's explanatory power.

 Overall recommendation

 This study addresses an important topic and provides a useful exploratory comparison of housing preferences and modular housing acceptance across four Indonesian cities. The authors have made meaningful improvements in the revised manuscript, particularly in the theoretical framing and acknowledgment of limitations.

 However, further clarification is needed regarding cross-city statistical comparisons, measurement consistency, reproducibility, and the interpretation of relative weights. The missing bootstrap confidence intervals, despite being identified as an implemented revision, are a particular concern.

 I would support the manuscript following revisions that address these methodological issues and more clearly distinguish exploratory findings from statistically supported conclusions. The study has value as an initial investigation of modular housing acceptance, but its contribution should be presented within the limits of the sampling strategy and analytical design.

Is the work clearly and accurately presented and does it cite the current literature?

Yes

If applicable, is the statistical analysis and its interpretation appropriate?

Partly

Are all the source data underlying the results available to ensure full reproducibility?

Partly

Is the study design appropriate and is the work technically sound?

Partly

Are the conclusions drawn adequately supported by the results?

Partly

Are sufficient details of methods and analysis provided to allow replication by others?

Partly

Reviewer Expertise:

Building Science and Environmental Design; Sustainable Architecture and Construction; Building Performance Analysis; Modular and Prefabricated Housing; Urban Planning and Housing Research; Computational Design and Environmental Visualization.

I confirm that I have read this submission and believe that I have an appropriate level of expertise to confirm that it is of an acceptable scientific standard, however I have significant reservations, as outlined above.

# Reviewer report: Reviewer response for version 2

**DOI:** 10.5256/f1000research.213861.r537442

**Published:** 2026-11-05

**Authors:** Rita Alfin

**Affiliations:** 1 University of Walisongo Gempol, Pasuruan, Indonesia

All revisions and clarifications requested have been satisfactorily addressed by the author. I have reviewed the revised manuscript and accepted all the changes.

Is the work clearly and accurately presented and does it cite the current literature?

Partly

If applicable, is the statistical analysis and its interpretation appropriate?

Yes

Are all the source data underlying the results available to ensure full reproducibility?

Partly

Is the study design appropriate and is the work technically sound?

Partly

Are the conclusions drawn adequately supported by the results?

Partly

Are sufficient details of methods and analysis provided to allow replication by others?

Partly

Reviewer Expertise:

Business administration, strategic management, consumer behavior, and market segmentation in the property and housing sector.

I confirm that I have read this submission and believe that I have an appropriate level of expertise to confirm that it is of an acceptable scientific standard.

# Reviewer report: Reviewer response for version 1

**DOI:** 10.5256/f1000research.204806.r527152

**Published:** 2026-10-29

**Authors:** Yanita Mila Ardiani

**Affiliations:** 1 Bina Nusantara University, Jakarta, Indonesia

Peer Review Result - Ethical Considerations The manuscript has adequately addressed the four fundamental aspects of ethical considerations in scientific publication, demonstrating a high standard of research integrity.

 1. Ethics - Respondent Protection: The authors have clearly elaborated the ethical approval process, informed consent procedures, and measures taken to ensure anonymity and confidentiality of respondents. The protection of vulnerable participants and the voluntary nature of participation have been explicitly stated, which reflects full compliance with ethical publication standards.

 2. Data Transparency: The study provides commendable transparency regarding data availability and research instruments. The authors have described the accessibility of datasets and questionnaires, enabling replicability and verification. This openness significantly strengthens the credibility and accountability of the research.

 3. Quality of Reporting: The reporting quality is excellent. The manuscript follows a recognized reporting guideline with systematic, coherent, and detailed presentation of methodology, results, and discussion. The logical flow and adherence to academic writing standards facilitate critical appraisal by readers

 4. Appreciation of Contributions: The contribution and acknowledgment section has been properly presented. All supporting parties, funding sources, and collaborators have been appropriately credited, avoiding any potential conflict of interest or omission of authorship. Overall Assessment: The ethical considerations are considered very good.

 Recommendation for Enhancement: However, for future improvement, it is suggested that the authors consider incorporating a visual mapping that illustrates a comparative analysis of satisfaction and dissatisfaction levels regarding the use of modular products across different cities. Such a geospatial visualization would not only enrich the analytical depth but also provide a more intuitive and comprehensive understanding of regional variations, user preferences, and contextual factors influencing product acceptance. This visual comparison would add significant value to the practical implications and policy relevance of the study.

Is the work clearly and accurately presented and does it cite the current literature?

Yes

If applicable, is the statistical analysis and its interpretation appropriate?

Partly

Are all the source data underlying the results available to ensure full reproducibility?

Yes

Is the study design appropriate and is the work technically sound?

Yes

Are the conclusions drawn adequately supported by the results?

Yes

Are sufficient details of methods and analysis provided to allow replication by others?

Yes

Reviewer Expertise:

The experimental design, including controls and methods, is adequate; results are presented accurately and the conclusions are justified and supported by the data.

I confirm that I have read this submission and believe that I have an appropriate level of expertise to confirm that it is of an acceptable scientific standard.

# Reviewer report: Reviewer response for version 1

**DOI:** 10.5256/f1000research.204806.r507479

**Published:** 2026-08-05

**Authors:** Rita Alfin

**Affiliations:** 1 University of Walisongo Gempol, Pasuruan, Indonesia

This manuscript examines factors influencing public acceptance of modular housing across four Indonesian cities (Jakarta, Surabaya, Sidoarjo, Malang) with distinct urban typologies. Using survey data from 300 respondents, the authors apply CFA to validate seven housing preference constructs and RWA to determine each construct's relative contribution to acceptance. Construction & Housing Quality emerges as the dominant predictor in most cities, except Sidoarjo where Financial Consideration ranks first. The authors propose a "typology-preference alignment" framework and offer practical implications for developers and policymakers.

 The manuscript is generally well-written and grounded in current literature, but the abstract contains an editorial inconsistency ("is" used instead of punctuation to separate findings) that should be corrected, and the theoretical framework (Figure 1) needs an accompanying conceptual narrative.

 The study design and RWA method are appropriate, but the per-city sample size (n=75) is small for city-level CFA/GOF evaluation and should be acknowledged as a limitation.

 Methodological detail is largely sufficient, though the full list of 38 measurement indicators should be provided as supplementary material for replication.

 Statistical interpretation is reasonable, but RWA weights are reported without confidence intervals, making it unclear whether cross-city differences are statistically meaningful. Several demographic-based interpretations are narrative rather than statistically tested and should be clearly labeled as tentative.

 The core conclusions are supported by the data, but the claimed novel contribution ("typology-preference alignment") needs stronger theoretical positioning, as it is currently based on a descriptive pattern from only four cases without significance testing.

 Points that must be addressed:

 1. Fix the editorial error in the abstract.

 2. Add conceptual narrative linking the theoretical framework to the constructs.

 3. Acknowledge the small per-city sample size as a limitation.

 4. Provide the full list of measurement indicators as supplementary material.

 5. Report bootstrap confidence intervals for RWA weights.

 6. Separate statistically tested findings from narrative interpretation.

 7. Strengthen the theoretical positioning of the proposed framework.

Is the work clearly and accurately presented and does it cite the current literature?

Partly

If applicable, is the statistical analysis and its interpretation appropriate?

Yes

Are all the source data underlying the results available to ensure full reproducibility?

Partly

Is the study design appropriate and is the work technically sound?

Partly

Are the conclusions drawn adequately supported by the results?

Partly

Are sufficient details of methods and analysis provided to allow replication by others?

Partly

Reviewer Expertise:

Business administration, strategic management, consumer behavior, and market segmentation in the property and housing sector.

I confirm that I have read this submission and believe that I have an appropriate level of expertise to confirm that it is of an acceptable scientific standard, however I have significant reservations, as outlined above.

# Response

**Published:** 2026-08-05

**Authors:** Alfandi Rizki Pratama

**Affiliations:** Brawijaya University Department of Civil Engineering, Malang, East Java, Indonesia

Dear Editor and Reviewer,

 Thank you for the constructive and detailed feedback on our manuscript, "Urban Housing Preferences and Public Acceptance of Modular Housing Across Different City Typologies in Indonesia." We appreciate the reviewer's careful reading and have revised the manuscript accordingly. Below we provide a point-by-point response to each comment. 

 1. Fix the editorial error in the abstract.

 We thank the reviewer for pointing this out. The Methods sentence in the Abstract listed the four study cities directly after the phrase "different urban typologies" without a separating punctuation mark. We have inserted a colon before the list of cities so the sentence now reads: "...in four cities with different urban typologies: Jakarta, Surabaya, Sidoarjo, and Malang, through a structured survey of 300 respondents..." We also checked the rest of the manuscript for the same construction to ensure consistency.

 2. Add conceptual narrative linking the theoretical framework to the constructs.

 We agree that Figure 1 required an accompanying narrative. We added a new paragraph at the end of the Theoretical Framework section explaining that the seven housing preference constructs are positioned as exogenous predictors with direct paths to Public Acceptance of Modular Housing, while urban typology functions as a contextual moderator that reshapes the relative strength of those paths rather than as an additional predictor. A short note has also been added to the Figure 1 legend referring back to this explanation.

 3. Acknowledge the small per-city sample size as a limitation.

 We agree that n = 75 per city is relatively small for city-level CFA and goodness-of-fit evaluation. We added this as a fourth limitation in the Conclusion, noting that the city-specific fit indices and RWA estimates should be interpreted as indicative rather than definitive, and that replication with larger city-level samples is recommended.

 4. Provide the full list of measurement indicators as supplementary material.

 We have compiled the complete list of the 38 measurement indicators, together with their source literature, as Supplementary Material (Appendix A), submitted alongside this letter. A reference to this appendix has been added in the Methods, and a new "Supplementary Material" statement has been added to the Back Matter.

 5. Report bootstrap confidence intervals for RWA weights.

 We agree that point estimates alone do not indicate whether cross-city differences in relative weights are statistically meaningful. We added a description of the bootstrap procedure (5,000 resamples per city, following Tonidandel and LeBreton, 2011) to the Methods, and we added 95% bootstrap confidence intervals to Table 8 alongside the existing point estimates, with an accompanying explanatory sentence in the Results describing how the intervals should be interpreted.

 6. Separate statistically tested findings from narrative interpretation.

 We agree that several demographic-based explanations in the Discussion could be read as statistically confirmed rather than interpretive. We added an explicit methodological caveat as a new paragraph at the start of the Discussion, stating that these explanations are narrative interpretations grounded in descriptive patterns rather than formally tested through moderation or interaction analysis. We also revised the wording of the individual demographic explanations throughout the Discussion to use tentative language.

 7. Strengthen the theoretical positioning of the proposed framework.

 We agree that the typology-preference alignment concept required stronger theoretical grounding. We expanded the relevant paragraph in the Discussion to situate the pattern within the person-to-environment fit tradition in housing research and clarified that it is an extension of existing preference-context theory rather than a replacement. We also added a qualifying statement in the Conclusion explicitly framing typology-preference alignment as a conceptual heuristic derived from four cities that requires confirmatory testing in future research, rather than a statistically validated typology.

 We believe these revisions have fully addressed the reviewer's comments and have strengthened the manuscript's methodological transparency, theoretical positioning, and reproducibility. We thank the reviewer again for the constructive suggestions, which have substantially improved the manuscript.

 Sincerely,

 Alfandi Rizki Pratama R, Ludfi Djakfar, Kartika Puspa Negara

 Department of Civil Engineering, Brawijaya University, Indonesia
